# Using Motive‐Alignment to Enhance Environmental Education for Youth: A Participatory Field Experiment

**DOI:** 10.1111/desc.70197

**Published:** 2026-04-23

**Authors:** Jenna Spitzer, Stathis Grapsas, Astrid Poorthuis, Lysanne te Brinke, Sander Thomaes

**Affiliations:** ^1^ Department of Psychology Utrecht University Utrecht The Netherlands; ^2^ Department of Education and Pedagogy Utrecht University; ^3^ Department of Psychology, Education and Child Studies Erasmus University Rotterdam Rotterdam The Netherlands

**Keywords:** adolescence, civic engagement, climate communication, environmental education, environmental efficacy, pro‐environmental behavior

## Abstract

We conducted a pre‐registered longitudinal field experiment to test whether educational activities inspired by sustainability motive‐alignment theory can boost the effectiveness of real‐world environmental education. Motive‐alignment interventions aim to encourage behavior change by linking new behaviors with goals that are salient to a target group (e.g., for youth, experiencing autonomy, gaining peer status, or contributing to society). First, we used participatory methods to develop two motive‐alignment inspired (MA‐inspired) environmental education activities with youth. We then randomly assigned 31 classes of vocational school students to receive a lesson including the MA‐inspired activities or an existing lesson about climate change. Ordinal and linear hierarchical Bayesian regression analyses showed that, as hypothesized, the MA‐inspired lesson (vs. the original lesson) increased students’ involvement in school eco‐teams and pro‐environmental voting intentions from pre‐lesson to post‐lesson and two‐week follow‐up. Exploratory analyses suggested that youth who received the MA‐inspired (vs. original) lesson did not feel more motive‐alignment during the lesson. However, they did report a heightened sense of environmental efficacy, indicating that the MA‐inspired lesson presented pro‐environmental engagement as more relevant to youth's motivation for societal contribution. These findings reveal the potential for motive‐alignment inspired educational activities—particularly those that tap into youth's motivation for societal contribution—to enhance real‐world environmental education.

**Summary:**

- We co‐developed activities with youth, inspired by the principles of sustainability motive‐alignment, to integrate into an existing lesson about climate change
- In a field experiment, the lesson including these activities (vs. the original lesson) increased students’ involvement in school eco‐teams and pro‐environmental voting intentions.
- Motive‐alignment inspired activities that foster environmental efficacy—thus aligning with youth's heightened motivation for contribution—can enhance the impact of environmental education in real‐world contexts.

## 1 Introduction

A main aim of environmental education is to impart to youth the motivation to take action on ecological challenges such as climate change (Batsa et al. 2025; Hungerford and Volk 1990). Though environmental education has the potential to empower youth engagement in sustainability transitions (UNESCO 2020; van de Wetering et al. 2022), its effectiveness among older youth is limited (Świątkowski et al. 2024). Theory and preliminary research suggest that educational approaches based on the principles of “motive‐alignment” (also called “values‐alignment”; Bryan et al. 2016; Galla et al. 2021) can strengthen the motivational impact of environmental education for youth. These approaches convey how pro‐environmental (i.e., sustainable, or “green”) engagement is relevant to youth's developmentally‐salient goals (Thomaes et al. 2023; van De Wetering et al. 2025). However, empirical evidence from real‐world school contexts is still lacking: Can motive‐alignment add to the effectiveness of existing environmental education programs? In this participatory, mixed methods study, we address this gap by conducting a pre‐registered longitudinal field experiment.

### 1.1 Engaging Youth through Environmental Education

The current generation of youth is one of the first to grow up with the widespread and tangible impacts of climate change. While youth cannot be held accountable for solving climate change, a problem for which they are minimally responsible, helping young people understand and contribute to its mitigation is critical to shaping a sustainable future. Indeed, the United Nations’ Sustainable Development Goals state that all youth should “acquire the knowledge and skills needed to promote sustainable development, including, among others, through education for sustainable development and sustainable lifestyles” (UNESCO 2020; United Nations 2015).

Supporting pro‐environmental engagement among youth is also developmentally opportune. Adolescence is a formative time of learning and growth, in which individuals’ longer‐term developmental trajectories take shape (Dahl et al. 2018). Rapid social and cognitive developments during adolescence, such as an increased capacity for perspective‐taking and a heightened need for developing meaning and purpose, can motivate youth to engage proactively with societal problems (Crone et al. 2024; Fuligni 2019). In addition, adolescents’ psychological flexibility and increased sensitivity to social justice can motivate them to be agents of change, as their central involvement in international climate movements illustrates (Cotovio 2023; Fritz et al. 2023). Around the world, youth have been frontrunners in global action (e.g., through their involvement in climate protests, spreading of pro‐environmental norms via social media, and bringing of lawsuits against (inter)national government bodies; Fritz et al. 2023; Irfan 2019).

As such, adolescence is a critical developmental stage for delivering environmental education. Such education helps youth to discover how they can have an impact on the environment—in both positive and negative ways—through their actions. Meta‐analytic evidence supports the potential for environmental education and similar interventions to enhance the pro‐environmental engagement of young people (with effect sizes ranging from small to large, depending on the measured outcomes; Świątkowski et al. 2024; van de Wetering et al. 2022). However, these effects are weaker in (older) adolescents as compared to children (Świątkowski et al. 2024). This is consistent with evidence from the broader field of behavior change education, which suggests that traditional school‐based programs tend to be ineffective in promoting (older) adolescents’ behavior change (Steinberg 2015; Yeager et al. 2018). To increase the effectiveness of behavior change education in adolescents, theory and preliminary research point to the potential of motive‐alignment approaches (Bryan et al. 2016; Galla et al. 2021; Thomaes et al. 2023).

### 1.2 Using Motive‐Alignment to Enhance Environmental Education

Motive‐alignment interventions promote behavior change in a target group by communicating to the target group that new behaviors allow them to realize goals they already consider important (Bryan 2021). Rather than trying to change what the target group values, this approach frames the promoted behaviors as opportunities to pursue their existing goals, thereby increasing the motivational relevance of these behaviors. Research has demonstrated the potential of this approach among diverse populations (e.g., adolescents and adults; Hecht et al. 2023; Yeager et al. 2018) and for diverse behaviors (e.g., promoting healthy eating, reducing social media use; Bryan et al. 2016, Bryan et al. 2019; Galla et al. 2021).

Motive‐alignment may be particularly well‐suited to strengthen environmental education for youth, as it leverages adolescents’ tendency to focus on short‐term, as opposed to long‐term, goals (Braams et al. 2015; Crone and Dahl 2012; Telzer 2016). Traditional programs encouraging behavior change often emphasize the long‐term benefits of the promoted behaviors. For example, environmental education programs often aim to increase pro‐environmental engagement by emphasizing the importance of preventing climate catastrophes from unfolding in the future (e.g., Greenpeace UK 2020). However, long‐term consequences can lack motivational force, particularly among youth (Thomaes et al. 2023; Yeager et al. 2018). To increase the personal appeal of pro‐environmental engagement, motive‐alignment approaches emphasize how such behaviors align with developmentally‐salient goals that tend to matter to youth in their daily lives, such as to experience autonomy (Crone and Fuligni 2020), gain peer status (Blakemore and Mills 2014; LaFontana and Cillessen 2010), or contribute to society (Fuligni 2019; Wray‐Lake and Ballard 2023). Though individuals across age groups can be motivated by these goals (Ryan 2023), physiological and environmental changes that occur during adolescence make these motivations salient to adolescents, in particular (Crone and Dahl 2012; Dahl et al. 2018; Van Den Bos 2013; Yeager et al. 2018). For example, recent research suggests that older adolescents and emerging adults, more so than other age groups, experience an imbalance between their motivation to contribute to society and their (limited) opportunities to make a societally‐relevant impact (Crone et al. 2024; Fuligni 2020; Te Brinke et al. 2022). By presenting pro‐environmental engagement as an opportunity for youth to realize salient goals, such as to contribute to addressing societal problems like climate change, motive‐alignment interventions can imbue pro‐environmental engagement with more personal, proximal appeal (Thomaes et al. 2023).

Motive‐alignment interventions can overlap with established approaches to encouraging pro‐environmental engagement (e.g., strengthening efficacy beliefs, setting social norms, or using autonomy‐supportive communication; Lavergne et al. 2010; Nolan 2021; van Valkengoed et al. 2022). For example, when seeking to appeal to youth's motivation for societal contribution, motive‐alignment interventions can emphasize that youth can make a meaningful impact through their pro‐environmental engagement, thus enhancing their sense of environmental efficacy. Similarly, when seeking to appeal to youth's motivation for peer status, motive‐alignment interventions may make use of social norm setting to signal that pro‐environmental engagement is respected and popular among youth's peers (van de Wetering et al. 2026). Rather than offering an alternative to these established approaches, motive‐alignment theory provides an additional lens for understanding why these approaches might be effective: because they imbue pro‐environmental engagement, which might otherwise be associated with distant benefits (e.g., preventing environmental catastrophes), with benefits that have more personal, proximal relevance (e.g., feeling impactful or fitting in with peers).

Initial studies conducted in controlled research settings demonstrate the potential for motive‐alignment to promote adolescents’ pro‐environmental engagement. For example, one experiment found that presenting pro‐environmental engagement as an opportunity for youth to stand up to companies that contribute to deforestation (thus tapping into a motivation for autonomy) increased their pro‐environmental behavior intentions and signing of an environmental petition (van De Wetering et al. 2025). Another experiment found that presenting sustainable foods as popular among youth's peers (communicating peer norms and, thus, tapping into a motivation for peer status) increased adolescents’ preference for more sustainable foods (van de Wetering et al. 2026). However, motive‐alignment approaches have not yet been evaluated when integrated into existing environmental education programs, implemented in real‐world educational settings. Here, we address this gap by testing whether adding a motive‐alignment inspired educational component strengthens environmental education.

### 1.3 Evaluating the Impact of Environmental Education

Youth can—and do—engage in environmentally‐impactful behaviors in diverse societal roles (e.g., as consumers, community members, citizens; Irfan 2019; Nielsen et al. 2021a; Ridder and Thomaes 2025). However, evaluations of environmental education (and psychologically informed environmental interventions, more generally) typically focus on their impact on consumer behaviors (e.g., purchasing choices, use of resources like water and electricity; Nielsen et al. 2021b; Ok and Kyung 2004; Zsóka et al. 2013). To broaden understanding of the effectiveness of environmental education, it is important to investigate how it shapes youth engagement in their diverse societal roles.

For instance, still little is known about whether environmental education supports pro‐environmental engagement in youth's roles as community members (e.g., by increasing their involvement in sustainability initiatives or groups) and as citizens (e.g., as future voters, by increasing their pro‐environmental voting intentions). School initiatives that give youth leadership roles in promoting sustainability both in and outside the school context enable youth to gain hands‐on experience shaping community values and pro‐environmental norms (Flanagan et al. 2019; Kiss et al. 2024; Lindemann‐Matthies et al. 2021). As a result, these programs present opportunities for youth to internalize pro‐environmental values, practice pro‐environmental behaviors, and develop a sense of environmental efficacy (i.e., the belief that they can, individually or collectively, contribute meaningfully to addressing climate change; Chen and Hsieh 2023; Cincera et al. 2023).

Regarding youth's role as future voters, supporting their development of pro‐environmental voting intentions is particularly opportune, as it is during adolescence that individuals’ critical consciousness, political identities, and norms around civic engagement emerge (Heberle et al. 2020; Rekker et al. 2017; Wray‐Lake and Ballard 2023). Even if, in most countries, youth only have a legal capacity to vote from age 18, supporting their development of pro‐environmental voting intentions is consequential. Youth voting intentions predict their future voting behavior (Eckstein et al. 2013), and early voting is predictive of long‐term voting engagement (Gerber et al. 2003; Plutzer 2002). Thus, shaping youth voting intentions can have lasting consequences. Furthermore, although most youth experience concern about climate change (United Nations Development Programme [UNDP] 2021), support for right‐wing, populist parties (that tend to oppose climate policies) is on the rise among youth (Foa and Mounk 2019; Henley and Sauer 2023; ProDemos n.d.). As public support for political parties invested in addressing climate change is essential for realizing sustainability transitions (Finnegan 2022; Wynes et al. 2021), it is critical to understand whether and how environmental education can motivate youth to contribute to environmental protection through their decisions as voters.

### 1.4 Present Research

We conducted a mixed methods study, using participatory, qualitative, and longitudinal field experimental methods. First, in collaboration with youth (Leask et al. 2019; Ozer and Piatt 2017), we developed educational activities inspired by the principles of motive‐alignment. This step allowed us to investigate what language and framing youth believed would best speak to the motives of youth their age. Next, we conducted a pre‐registered, longitudinal field experiment to test whether a lesson including these motive‐aligned‐inspired (MA‐inspired) lesson components (versus the original lesson), increased youths’ (1) engagement in school eco‐teams (i.e., interest in joining a group of students to design a greener schoolyard in collaboration with a landscape architect), and (2) pro‐environmental voting intentions (i.e., interest in voting for political parties that aim to address climate change). Finally, we conducted interviews with youth who participated in their schools’ eco‐teams to gain additional insight into the drivers of their engagement.

We conducted this research among Dutch vocational school students in collaboration with two societal partners: the Youth Climate Movement (i.e., *Jonge Klimaatbeweging*), the biggest youth‐led climate organization in the Netherlands, and Trees for Al, a non‐profit organization sponsoring the Trees for Schools program. As part of the Trees for Schools program, vocational students in participating Dutch schools received (1) a lesson about climate change, delivered by members of the *Youth Climate Movement*; (2) support in creating school‐based eco‐teams, to design greener schoolyards; and (3) assistance in realizing the eco‐teams’ plans for a greener schoolyard, through a Tree Planting Day. The Youth Climate Movement and Trees for All decided to direct this programming towards youth in vocational education, as these youth tend to have fewer opportunities for pro‐environmental engagement compared to youth in other, theoretical or pre‐academic forms of education (Hoekstra et al. 2024; Vadén et al. 2025). Moreover, youth in vocational education tend to be underrepresented in research (Fakkel et al. 2020), and are highly relevant from a sustainability perspective, in particular. In their professional futures, these youth are expected to play a major role in enabling sustainability transitions (e.g., European Commission 2024; UNESCO‐UNEVOC n.d.). At the same time, research suggests that youth in vocational education tend to hold higher levels of climate change skepticism and express less support for climate policies compared to youth in pre‐university education (de Graaf et al. 2023; Vadén et al. 2025). Developing effective environmental education for vocational school students is thus critical.

## 2 Method

### 2.1 Ethics and Open Science Statement

The ethics committee of Utrecht University approved this research. We preregistered the study hypotheses, methods, and analysis plan prior to data collection, and the data and code needed to reproduce our results are available on the Open Science Framework (https://osf.io/jc8e3).

### 2.2 Intervention Development with Youth

#### 2.2.1 Participants and Procedure

To develop the MA‐inspired activities, we (1) conducted a co‐creation session with youth to generate ideas for the activities, (2) trialed the newly‐developed activities, and (3) piloted the full lesson integrating the newly‐developed activities into an original lesson about climate change. The youth who participated in the intervention development process were predominantly ethnically Dutch and from middle class, urban and suburban communities. In the co‐creation session, organized at a community center, participants (*N* = 11; 36.4% female; *M*_age_ = 22.75, *SD*_age_ = 4.32) brainstormed about activities that could communicate that pro‐environmental engagement enables youth to express their autonomy, gain respect from their peers, and contribute to society. We trialed the two MA‐inspired activities discussed in this session with two classes of vocational school students (*N* = 26; 50% female; *M*_age_ = 18.15, *SD*_age_ = 2.89). We then updated the activities according to student feedback and piloted the full‐length lesson with another class of vocational school students (*N* = 18; 61.1% female; *M*_age_ = 18.10, *SD*_age_ = 1.52). We again used student feedback to create the final versions of the activities (see Results section for the main take‐aways from this co‐development process).

### 2.3 Longitudinal Field Experiment

#### 2.3.1 Participants and Procedure

Participants were 517 youth (*M*_age_ = 17.82, *SD*_age_ = 2.29; 57.45% female, 19.34% male, 23.21% not reported; 59% Dutch, 14.12% bi‐ethnic, 6.19% other, 20.70% not reported; predominantly from middle class, urban and suburban communities). We recruited youth from three vocational schools that agreed to participate in the Trees for Schools program. Like other vocational schools in the Netherlands, these schools offer training in diverse vocations (e.g., baking and pastry, hospitality management, construction). The schools selected which classes would take part in the Trees for Schools program (i.e., receive a lesson about climate change and have an opportunity to join the eco‐teams), based on practical considerations (e.g., scheduling constraints). At each of the three schools, approximately 10 classes (31 in total) received a lesson about climate change during the school day, in place of a regular class. At the end of the lesson, the students learned about the Trees for Schools program and their opportunity to join the eco‐teams.

**FIGURE 1:**
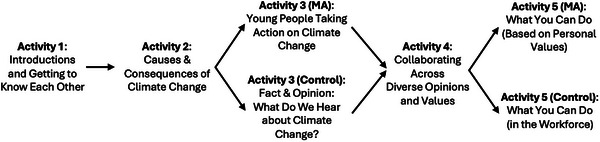
Overview of the control and MA‐inspired lessons about climate change. *Note*: MA = MA‐inspired lesson; Control = original lesson.

Data collection took place from fall 2023 to winter 2024. Classes were randomly assigned to receive either the original (i.e., active control; *n* = 15 classes) or MA‐inspired (i.e., intervention; *n* = 16 classes) lesson about climate change. Both lessons lasted approximately 1.5 h and included a total of five activities, differing with respect to two of the activities (see Figure 1 for a visual overview of the lessons and see the supplementary material for a description of the activities included in each lesson).

In the MA‐inspired lesson, the third activity involved an in‐class conversation about why youth are increasingly taking action on climate change, and the fifth activity invited students to reflect individually and write about how they can contribute to sustainability efforts in ways that suit who they are. These activities aimed to align with youth motives for autonomy, peer status, and societal contribution by emphasizing (1) how youth can enact their values and express who they are through pro‐environmental engagement; (2) that youth are increasingly committed to pro‐environmental engagement; and (3) that youth can have an impact on those around them and society at large through pro‐environmental engagement. For example, in the third activity, participants reflected on the high rates of pro‐environmental engagement among youth (communicating peer norms, to tap into a motivation for peer status) and discussed why their generation is particularly well‐suited to address environmental problems (signaling their potential to make an impact through autonomous action, to tap into motivations for societal contribution and autonomy). In the fifth activity, participants reflected on why youth who take action on climate change can gain respect from those around them (to tap into a motivation for peer status), and how they can translate their personal strengths and values into diverse forms of pro‐environmental engagement in their daily lives (to tap into motivations for societal contribution and autonomy).

In the control condition, the third activity involved identifying the difference between a fact and an opinion, and the fifth activity involved a reflection on how their future occupations could be relevant to sustainability transitions. These original activities were not designed to be misaligned with motivations for autonomy, peer status, and societal contribution, but they were less explicitly supportive of these goals. For example, in the third activity of the original lesson, teachers did not communicate peer norms about how youth tend to relate to climate and environmental issues. In the fifth activity of the original lesson, students only reflected on their future careers, creating temporal distance between their current lives and their ability to contribute.

All lessons were delivered by members of the *Youth Climate Movement*, who were trained by our research team in how to teach both lessons. The “guest teachers” and school coordinators were kept blind to the study aims and hypotheses, and the same “guest teachers” were assigned to teach both lessons over the course of the study.

#### 2.3.2 Measures

##### 2.3.2.1 Eco‐Team Engagement

All students (*N* = 517) who participated in a lesson about climate change reported directly following the lesson (i.e., post‐test) whether they were interested in learning more about joining the eco‐team (0 = *no*, 1 = *yes*). Approximately two weeks following the lesson (i.e., two‐week follow‐up), school coordinators contacted the students who expressed an initial interest to ask if they were still interested in participating (0 = *no*, 1 = *yes*). At four‐week follow‐up, school coordinators again contacted students who expressed an interest to ask if they would participate in the first eco‐team meeting (0 = *no*, 1 = *yes*). We computed a total score representing the ordered degree of participants’ eco‐team interest and engagement: 0 (*not interested after lesson)*, 1 (*interested after lesson*), 2 (*interested at two‐week follow‐up*), and 3 (*signed‐up at four‐week follow‐up for the first eco‐team meeting*). The total score thus represents participants’ ordered progression of eco‐team interest and engagement, as later‐stage indicators were offered only to participants who had expressed an interest at the prior stage(s). In one of the three participating schools, students received a form of course credit (i.e., “citizenship points”) for participating in the eco‐team. Importantly, the school did not inform students that they would receive credit for participating until after the initial lesson. Thus, all participants who joined the eco‐team expressed an interest in doing so before they were offered course credit.

##### 2.3.2.2 Pro‐Environmental Voting Intentions

To measure pro‐environmental voting intentions, we used three items developed for the purposes of this study (i.e., *How interested are you in voting for a political party that wants to address climate change?*, *How important would climate change be to you if you were voting?*, and *How sure are you that you would want to vote for a political party that aims to address climate change?*). Using a sliding scale from 0 (*not at all*) to 100 (*very much*), participants reported their responses in in‐class online surveys one week before the intervention (pre‐test; *N* = 416), directly following the lesson (post‐test; *N* = 381), and two‐weeks following the lesson (two‐week follow‐up; *N* = 395). Across measurement waves, 490 participants (94.78%) reported on their pro‐environmental voting intentions at least once. We computed the mean of all items (Cronbach's α = 0.90, 0.93. and 0.92 at pre‐test, post‐test, and two‐week follow‐up, respectively).

##### 2.3.2.3 Exploratory Measures

As part of the post‐test survey, we included measures to explore whether the co‐created activities aligned pro‐environmental engagement with youth's motivations to express autonomy, gain peer status, or make a societal impact (see supplementary material for details on the measures).

##### 2.3.2.4 Follow‐Up Interviews with Eco‐Team Participants

At the end of the Trees for Schools program, 5–6 months following the lesson about climate change, the first author interviewed youth who joined the school eco‐teams and were present for the “Tree‐Planting Day” (i.e., the final activity of the eco‐teams, in which students planted new plants and trees according to their design with the landscape architect; *N* = 16, with at least one student represented from each of the three schools; *M*_age_ = 18.45, *SD*_age_ = 2.87, 93% female). These brief (approximately 5 min), semi‐structured interviews took place in‐person (*n* = 11) or online (*n* = 5). The interview included questions about participants’ motivations for participating in the eco‐teams, their reflections on the lesson about climate change, and their sense of how they had (or had not) changed through their participation in the eco‐teams. These questions were selected to understand the drivers and consequences of youth pro‐environmental engagement. At the end of the interview, participants received €10 to thank them for their time.

Interviews were transcribed and translated (i.e., from Dutch into English), and the transcripts were analyzed side‐by‐side by the first author, who speaks both languages. The first author is a developmental psychologist with a research focus on youth responses to climate and environmental problems. As an adult researcher studying youth's experiences, she recognizes the generational power dynamics inherent in this work. Her prior research has emphasized young people's agency, which may shape how findings are interpreted. To account for these influences, analytic decisions were discussed within the research team, and interpretations were grounded closely in the data.

We conducted inductive thematic analysis with the aim of describing patterns in participants’ responses without having a preconceived focus (Braun and Clarke 2006, 2019). Our general guiding questions were: *What personal and contextual factors drive youth pro‐environmental engagement?* and *What, if anything, do youth take away from pro‐environmental engagement?* We approached qualitative analysis an active, subjective, and reflexive process.

Following Braun and Clarke (2006), the first author generated initial codes, re‐evaluated and grouped the codes into higher‐order codes, and then used the codes to define three overarching themes. The movement from codes to the broader definition of themes involved an iterative process of sorting the codes, consulting the coded data associated with the grouped codes, and refining the themes to reflect the data across codes. Using the first author's logbook, one of the co‐authors ([BLINDED FOR REVIEW]) reviewed the qualitative analysis process following the recommended procedure of Akkerman et al. (2008), to confirm the visibility, comprehensibility, and acceptability of the analyses and their resulting conclusions.

#### 2.3.3 Statistical Approach

To check if random assignment to the experimental conditions was successful, we evaluated whether conditions differed by participants’ gender, age, school, or pro‐environmental voting intentions at pre‐test. To check the potential influence of missing data, we also evaluated associations between condition and missing data on pro‐environmental voting intentions at pre‐test, post‐test, and follow‐up (there was no missing data regarding eco‐team engagement). Following our pre‐registration, we evaluated associations between participants’ environmental outcomes and their gender, age, school, and positive perceptions of the guest teachers at post‐test, to determine if these variables should be included as covariates in sensitivity analyses.

To test our hypothesis (i.e., that youth in the MA‐inspired condition would exhibit more pro‐environmental engagement than youth in the control condition), we fit multilevel mixed‐effects models. Such models account for the hierarchical data structure, with observations nested in participants (for pro‐environmental voting intentions), and participants nested in classrooms. To evaluate condition differences in eco‐team engagement, we conducted ordinal regression analysis. To evaluate condition differences in change in participants’ pro‐environmental voting intentions over time, we conducted linear regression analysis evaluating the interaction effects between condition and time (at post‐test and two‐week follow‐up). We also conducted exploratory (i.e., non‐preregistered) analyses investigating the impact of the MA‐inspired activities on measures of the targeted motivations.

Following our pre‐registration, we ran our confirmatory analyses using both Bayesian and frequentist approaches, including all observations of our primary outcome variables. For the Bayesian analyses, also following our pre‐registration, we used Bayesian informative hypothesis testing to evaluate evidence for the model reflecting our hypothesis (i.e., *H_+_* = pro‐environmental engagement in the MA‐inspired condition > pro‐environmental engagement in the control condition) compared to its complement (i.e., *H_c_* = pro‐environmental engagement in the MA‐inspired condition ≤ pro‐environmental engagement in the control condition; van Lissa et al. 2021). We computed Bayes Factors (BFs, indicating the relative support for the hypothesized model) and Posterior Model Probabilities (PMPs, indicating the probability of the hypothesized model given the data). A BF > 1 indicates support for *H_+_*, whereas a BF < 1 indicates support for *H_c_*_._ The greater the BF is than 1, the stronger the evidence for *H_+_*, whereas the less the BF is than 1, the stronger the evidence for *H_c_*. A PMP ranges from 0 to 1, with higher values indicating a higher probability that the model represents the data. As Bayesian methods produce more accurate estimates of uncertainty for hierarchical models (Kruschke and Vanpaemel 2015), we present the results of our Bayesian analyses in the main text and include the results of our frequentist analyses in the supplementary material.

## 3 Results

### 3.1 Intervention Development with Youth

Youth feedback helped us develop the framing and content of the MA‐inspired activities about climate change. For example, one of the activities initially emphasized that “there is no debate” about climate change being a real problem to communicate that peers think that climate change is a serious problem that needs to be addressed. Though this approach to communicating climate consensus has been found to be effective among adults (Većkalov et al. 2024), participants in the co‐creation session were critical of this language, as they felt it brought their attention to where debate remains. The final version of the lesson instead communicated that youth are aware of the consequences of climate change even more so than other generations, and that they are therefore highly motivated to do something about it. Youth feedback also helped shape the questions included in the final lesson to prompt reflection. For example, rather than encouraging participants to think about their values and connect them to their pro‐environmental engagement—an approach that participants found difficult and unclear—the final activity asked more direct and actionable questions (e.g., *How can you help create a more sustainable world in a way that suits who you are?*).

### 3.2 Longitudinal Field Experiment

#### 3.2.1 Preliminary Analyses

Table 1 presents descriptive statistics for the primary outcome variables. Conditions did not differ with respect to participants’ gender, age, school, or baseline pro‐environmental voting intentions (*p*s > 0.310), indicating successful randomization. In addition, conditions did not differ with respect to missing data on pro‐environmental voting intentions at pre‐test, post‐test, or two‐week follow‐up (*p*s > 0.246). Participants’ eco‐team engagement was associated with older age (*p* = 0.040), and their pro‐environmental voting intentions differed by school and were associated with older age and participants’ positive perceptions of the guest teachers (*p*s < 0.041; see supplementary material). We thus conducted additional sensitivity analyses to evaluate the robustness of our primary results when including these variables as covariates. Interclass correlations indicated that differences among classes accounted for only 6.95% of the variance in eco‐team participation and 5.31% of the variance in pro‐environmental voting intentions, suggesting that variation occurred primarily at the individual (not class) level.

**TABLE 1:** Descriptives for the primary outcome variables for the full sample and by condition.

Variable	Full sample	Control condition	MA condition
Eco‐team engagement^a^			
0 Frequency (%)	409 (79.11)	199 (81.56)	210 (76.92)
1 Frequency (%)	74 (14.31)	33 (13.52)	41 (15.02)
2 Frequency (%)	11 (2.13)	3 (1.23)	8 (2.93)
3 Frequency (%)	23 (4.45)	9 (3.69)	14 (5.13)
Pro‐env voting intentions			
Pre‐test *M* (*SD*)	51.69 (23.27)	51.83 (24.01)	51.56 (22.64)
*N*	416	196	220
Post‐test *M* (*SD*)	55.95 (23.18)	54.4 (22.61)	57.25 (23.62)
*N*	381	174	207
Follow‐up *M* (*SD*)	52.62 (23.18)	50.88 (22.77)	54.16 (23.49)
*N*	395	186	209

*Note*: Pro‐env voting intentions = pro‐environmental voting intentions. MA Condition = motive‐alignment.

^a^At each measurement wave, the *N*s for eco‐team engagement were 517 for the full sample, 244 for the control condition, and 273 for the motive‐alignment condition. 0 = not interested at post‐test, 1 = interested at post‐test, 2 = interested at two‐week follow‐up, and 3 = signed‐up for first meeting of eco‐team at four‐week follow‐up.

#### 3.2.2 Confirmatory Analyses

##### 3.2.2.1 Eco‐Team Engagement

**FIGURE 2:**
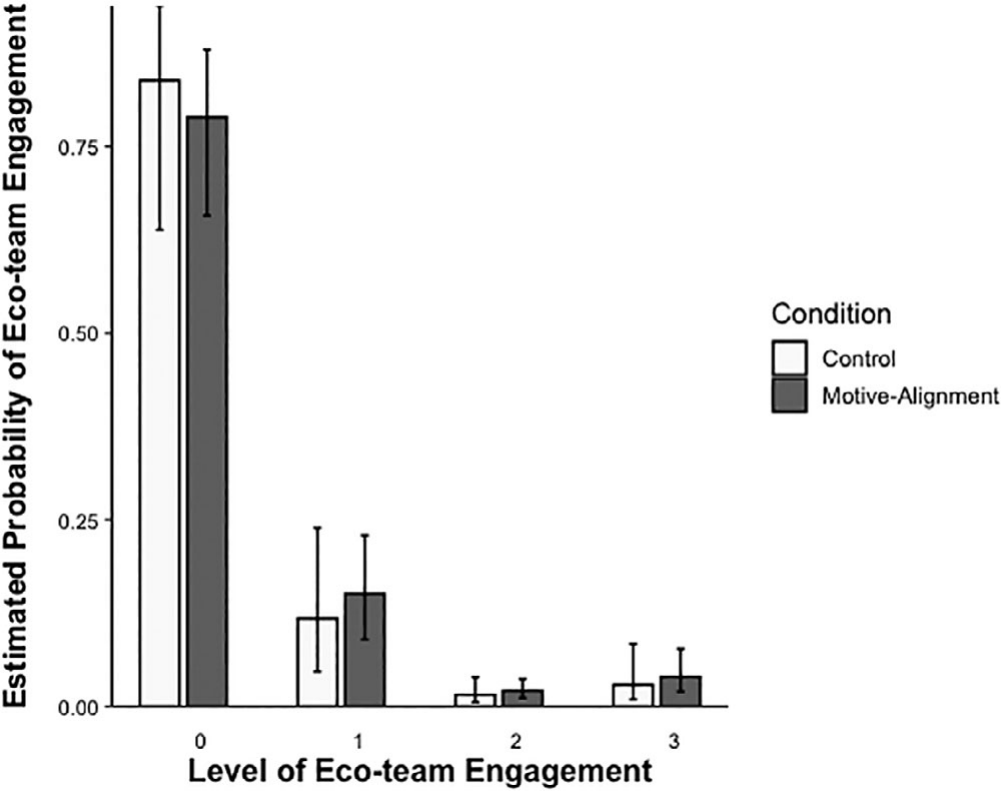
Estimated eco‐team engagement by condition*. Note*: The values per level of eco‐team engagement are: 0 = not interested at post‐test, 1 = interested at post‐test, 2 = interested at two‐week follow‐up, and 3 = signed‐up for first meeting of eco‐team at four‐week follow‐up.

Youth in the intervention condition exhibited greater eco‐team interest and engagement compared to youth in the control condition (see Table 2 and Figure 2; *H_+_* BF = 6.454; PMP = 0.866). For example, in the MA‐inspired condition, fewer participants were *not* interested in learning more about the eco‐team directly following the lesson (77.52% [95% CI = 65.00%–86.00%]), compared to the control condition (82.74% [95% CI = 63.06%–93.08%]). In the MA‐inspired condition, more youth also persisted in their interest and ultimately joined the eco‐teams (4.46% [95% CI = 2.20%–8.80%]), compared to the control condition (3.25% [95% CI = 1.04%–9.64%]). Sensitivity analyses including age as a covariate supported this same conclusion (see supplementary material).

**TABLE 2:** Bayesian mixed‐effects ordinal regression: Eco‐team engagement by condition.

Effect	Estimate	*Est. error*	95% CI	
			*LL*	*UL*
Fixed effects				
Intercept [0|1]	1.57	0.23	1.14	2.03
Intercept [1|2]	2.94	0.27	2.42	3.51
Intercept [2|3]	3.40	0.30	2.82	4.02
Condition	0.33	0.30	−0.27	0.92
Random effects				
Class ^a^	0.52	0.20	0.12	0.92

*Note*: The intercepts are thresholds representing the log‐odds of being in a certain category of the ordered outcome vs. any lower category (i.e., Intercept 0|1 = odds of expressing no interest in eco‐team at post‐test (0) vs. expressing interest in eco‐team at post‐test (1); Intercept 1|2 = odds of expressing interest at two‐week follow‐up (2) vs. being in category 0 or 1). Condition 0 = control; 1 = motive‐alignment.

Abbreviations: CI = confidence interval; *LL* = lower limit; *UL* = upper limit.

^a^Number of observations at the class level = 31.

##### 3.2.2.2 Pro‐Environmental Voting Intentions

**FIGURE 3:**
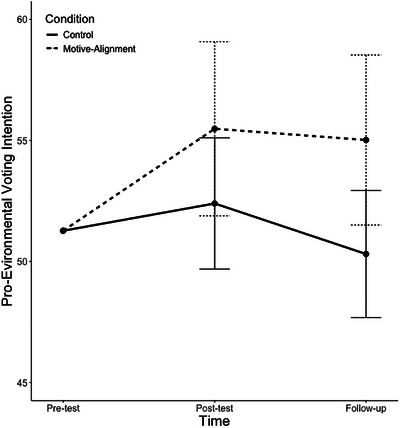
Estimated change in pro‐environmental voting intentions over time by condition. *Note*: Participants reported on pro‐environmental voting intentions at pre‐test (one week before the lesson about climate change), post‐test (directly following the lesson), and at two‐week follow‐up.

Youth in the MA‐inspired condition exhibited a greater increase in pro‐environmental voting intentions over time, compared to youth in the control condition (see Table 3 and Figure 3; H_+ Time1 *__Condition_ BF = 272.973; PMP = 0.996; H_+ Time2 *__Condition_ BF = 272.973; PMP = 0.996). Directly following the lesson, participants’ estimated pro‐environmental voting intentions were 73.49% [95% CI = 69.59%–77.05%] in the MA‐inspired condition and 67.41% [95% CI = 62.94%–71.61%] in the control condition (compared to 69.94% and 71.89% at baseline, respectively). Two weeks following the lesson, participants’ estimated pro‐environmental voting intentions were 76.29% [95% CI = 72.68%–79.58%] in the MA‐inspired condition and 65.76% [95% CI = 61.22%–70.26%] in the control condition. Sensitivity analyses including age, school, and the favorability of participants’ perceptions of the guest teachers as covariates supported this same conclusion (see supplementary material).

**TABLE 3:** Bayesian mixed‐effects linear regression: Change in pro‐environmental voting intentions over time by condition.

Effect	Estimate	Est. Error	95% CI	
			*LL*	*UL*
Fixed effects				
Intercept	51.28	1.49	48.29	54.20
Time 1	1.14	1.38	−1.56	3.81
Time 2	−0.94	1.36	−3.62	1.70
Time 1 ^*^ condition	4.20	1.83	0.61	7.78
Time 2 ^*^ condition	3.73	1.81	0.20	7.28
Random effects				
Individual ^a^	18.71	0.78	17.24	20.27
Class ^b^	5.37	1.61	2.16	8.65

*Note*: CI = confidence interval; *LL* = lower limit; *UL* = upper limit. Time 1 = dummy coded to represent post‐test, compared to pre‐test. Time 2 = dummy coded to represent follow‐up, compared to pre‐test. Condition 0 = control; 1 = MA‐inspired.

^a^Number of observations at the individual level = 490.

^b^Number of observations at the class level = 31.

#### 3.2.3 Exploratory Analyses

Directly following the lesson, youth in the MA‐inspired condition did not report feeling more motive‐alignment during the lesson than youth in the control condition. However, they did report a heightened sense of environmental efficacy compared to youth in the control condition. The MA‐inspired lesson thus presented pro‐environmental engagement as a more effective means for youth to make an impact, aligning pro‐environmental engagement with the targeted motivation of societal contribution (see supplementary material for detailed results).

### 3.3 Interviews with Eco‐Team Participants

We defined three overarching themes from the interviews, which appeared equally relevant to eco‐team participants from both conditions: (1) youth need opportunities for pro‐environmental engagement to be able to turn latent, pre‐existing motivations into action; (2) presenting pro‐environmental engagement as an opportunity to create a legacy (i.e., in the form of a more beautiful school garden) is motivating for youth; and (3) extrinsic reasons for pro‐environmental engagement (e.g., study credits) may limit the extent to which youth derive personal meaning from such engagement.

Regarding the first theme, many youth who joined the eco‐teams already seemed to view themselves as people who care about and want to support the natural environment. Rather than establishing a new desire to do something for the environment, the lesson about climate change helped these youth discover ways in which they can turn their existing pro‐environmental motivations, sometimes inspired by their parents or other family members, into action. As one participant (18‐year‐old female) said: “Without the class, I wouldn't have known this [program] was a thing for students, so in that sense it definitely helps”. Another participant (female, age not reported) reflected on how the lesson spoke to something that was already “in them”, even if it wouldn't speak to something that was in all students. Participants’ responses illustrate that youth pro‐environmental engagement depends upon a constellation of psychological and contextual factors (Steg and Vlek 2009): Youth not only need to be motivated to address environmental problems, but they also need to be aware of, and have access to, opportunities to get involved in addressing these problems. The interviews thus point to the importance of making opportunities for environmental engagement available to youth, especially as such engagement may further deepen and reinforce itself (e.g., “Now I know how to plant trees or plants…. I'm also going to plant plants now with my mom” [19‐year‐old female]).

Many participants also expressed that they were motivated by the idea of creating a legacy—a lasting contribution to their school that others can see, and that they can be proud of. As one participant (26‐year‐old male) explained, “I also just thought it would be fun to work in a group and still leave a little bit of your impression here at school, for when you leave again”. Notably, the idea of creating a legacy taps into core youth motivations for autonomy, status, and societal contribution: youth create a legacy, by making a positive impact (contribution) through their self‐directed (autonomous) actions, for which they will be remembered and viewed positively (status). Some participants also expressed their satisfaction at having made a positive impact through their efforts, suggesting that fulfilling these motives through pro‐environmental engagement can reinforce the value and benefit of such engagement. For example, one participant (19‐year‐old female) said: “We will actually finish this school and leave, but the little trees and plants that were planted will always grow here. So now I feel really… Yeah, I'm really proud of myself now too. Maybe next year, I'll come and see how it goes. So I really like that. I'm happy with that”. Participants also thought that demonstrating the lasting impact of their actions could motivate their peers to get involved in the future, for instance, by “showing pictures of the beginning with us and what it looks like now. That you also just really see that you're going to do something” [female, age not reported].

We identified another theme by considering participants’ motivations for joining the eco‐teams along with their sense of having been impacted by the experience. Students from the one school that offered course credit for participating in the eco‐team consistently mentioned course credit as a central reason for their engagement, even though these students expressed an interest in joining the eco‐team *before* they knew they could get credit for doing so. For example, students said they participated in the eco‐team “just for the citizen points” [Participant 13]) or because “we got citizen points for it. And citizenship points you just have to have for your education” [Participant 7], and “just for the citizen points” [Participant 13]). Interestingly, these participants also seemed to feel less impacted by the experience of participating: “No, actually no…. [how I think about climate change] is still the same” and “I don't necessarily think [I changed], but it was a lot of fun”, respectively. On the other hand, participants from other schools mentioned more intrinsic reasons for participating, and they also seemed more inclined to mention ways in which the program had impacted them. For example, one student [21‐year‐old female] said: “What I really liked is that with the school together, so with classmates and others, that we could really take care of it ourselves. That you had a little bit of your own direction, to bring new life to a courtyard” [21‐year‐old female], (e.g., “I do think [I have been] a little affected and a little changed…I was always in favor of nature anyway, but I'm a little bit more into it now…for example, [in] the forest, I don't just take it for granted, but really enjoy it”. These interviews thus suggest that how and why youth are motivated to engage in environmental initiatives may shape what they take away from such experiences.

## 4 General Discussion

In this participatory, mixed methods study, we tested whether adopting a developmentally‐informed approach to designing environmental education for youth—that is, motive‐alignment—can boost its capacity to encourage pro‐environmental engagement. We found that integrating activities inspired by the principles of motive‐alignment into an existing lesson about climate change increased students’ pro‐environmental engagement in their school communities (i.e., their likelihood of joining school eco‐teams) and as citizens (i.e., their pro‐environmental voting intentions). Thus, our study demonstrates that educational activities designed to speak to psychological motives relevant to youth can strengthen the effectiveness of real‐world environmental education—particularly when they convince youth that, through their pro‐environmental engagement, they can make a difference to the world around them.

### 4.1 Implications for Theory and Practice

For the first time, this research demonstrated that adopting a motive‐alignment approach to designing educational activities can boost the effectiveness of environmental education as implemented in a real‐world context (i.e., an existing lesson about climate change delivered in vocational schools). Youth who participated in the motive‐alignment inspired lesson, compared to those who participated in the original lesson, did not report experiencing more motive‐alignment during the lesson. We speculate that this finding may reveal a limitation of the co‐developed activities, or alternatively, a limitation of the study‐developed measure of state motive‐alignment to capture subtle changes in youth perceptions of pro‐environmental engagement. Still, the motive‐aligned inspired lesson did enhance youth's sense of environmental efficacy, indicating that it successfully presented pro‐environmental engagement as relevant to the targeted motivation for societal contribution. Thus, our research builds on prior work illustrating the importance of environmental efficacy for pro‐environmental engagement (Bradley et al. 2020; Jugert et al. 2016; van Valkengoed et al. 2022). It also shows that, to strengthen the motivational force of environmental education, educators may emphasize that pro‐environmental engagement is an effective opportunity for youth to make a positive impact on society.

Co‐developing the motive‐alignment inspired activities with youth helped us translate motive‐alignment theory into educational practice. In particular, the co‐creation session and pilot lessons with youth showed that activities intended to support motive‐alignment also risk provoking reactance (i.e., an aversive response to a sense that one's freedom is being threatened; Van Petegem et al. 2015). This risk, though ironic, is understandable. If youth experience activities as being normative (e.g., implying that youth *should* respect other youth who support sustainability) or restrictive (e.g., implying that youth *must* adopt a certain perspective on climate change), then such activities may backfire to the extent that they limit youth's sense of autonomy. Including youth in the intervention development process can help reduce this risk by providing insight into the language that youth find motivating, but not overbearing (Ozer and Piatt 2017). Indeed, even if science‐based guidelines for developing autonomy‐supportive interventions are available (Reeve 2016), how youth experience particular framing is likely generationally and geographically specific (Toenders et al. 2024).

The interviews with youth who participated in the eco‐teams can also inform theory and practice. For example, they illustrated that many youth experience an imbalance between their motivation to contribute to society and the opportunities they experience to make an impact (Fuligni 2020; Te Brinke et al. 2022). This reality calls upon educators and policymakers to create opportunities for youth to contribute to sustainability transitions, and to tap into adolescents’ burgeoning interest in becoming impactful members of society (Crone et al. 2024; Fuligni 2019). The interviews also indicated the potential benefit of presenting pro‐environmental engagement as an opportunity for youth to create a legacy, a goal that aligns simultaneously with youth motivations for autonomy, status, and societal contribution. Future interventions may thus benefit from including activities that adopt this framing (Zaval et al. 2015). In addition, the interviews indicated that extrinsic reasons for pro‐environmental engagement (e.g., course credit) may limit the extent to which youth extract personal meaning from such engagement. This finding suggests that externalized motivations for pro‐environmental engagement tend to be more fleeting or superficial, thus illustrating a potential downside of offering extrinsic rewards for engaging in sustainability initiatives. This idea accords with Self‐Determination Theory and literature on extrinsic motivation, more generally (Deci et al. 1999; Morris et al. 2022; Spitzer et al. 2024).

### 4.2 Strengths, Limitations, and Future Directions

By integrating activities inspired by motive‐alignment principles into an existing environmental education intervention, this research demonstrated the potential for this approach to enhance current environmental educational practice. We involved youth in the intervention development process, and we conducted this research in a particular subset of youth (i.e., vocational school students), who are highly relevant from a sustainability perspective (e.g., European Commission 2024; UNESCO‐UNEVOC n.d.), though they tend to be underrepresented in research (Fakkel et al. 2020).

Still, this study has limitations. First, the MA‐inspired lesson was designed to align pro‐environmental engagement with motivations known to be important to youth (i.e., for autonomy, peer status, and societal contribution; Fuligni 2019; Thomaes et al. 2023; Yeager et al. 2018), but we did not verify the relevance of these motivations among the particular youth in our study. Future research may develop more targeted (and, potentially, more effective) interventions by identifying goals that are most salient to individuals in the particular sample, and designing interventions that align targeted behaviors with those identified goals. Relatedly, as we did not measure participants’ personal motivations at the outset of the study, we were unable to evaluate whether the motive‐alignment inspired lesson encouraged more engagement among those youth who were more strongly motivated to contribute to society. Measuring participants’ personal motivations, as well as their perception of how much intervention materials include content related to the targeted motivations, could help establish the extent to which motive‐alignment serves as the underlying mechanism that drives behavior change.

Second, though the motive‐alignment inspired lesson was designed to speak broadly to youth motivations for autonomy, peer status, and societal contribution, we found evidence that the co‐developed activities enhanced participants’ sense of environmental efficacy, specifically (Damon et al. 2019; Fuligni 2009, 2020). We hope future research will develop and evaluate lesson components that more broadly speak to all targeted youth motivations (including those for autonomy and peer status), as these motivations may be powerful levers for action (Bryan et al. 2019; van de Wetering et al. 2026).

Third, we evaluated the impact of two relatively brief MA‐inspired activities (i.e., together taking about 30 min) integrated into a single climate change lesson: a relatively subtle intervention. Future research may reveal the short and longer‐term consequences of integrating MA‐inspired techniques more extensively, for instance, spread throughout an environmental education curriculum. In addition, we found intervention effects with respect to participants’ pro‐environmental voting intentions, which overlap with, but may differ from, actual voting behavior (Enamorado and Imai 2019). Future work could build on these findings by measuring actual voting behavior (e.g., using exit poll methodologies) and other age‐relevant measures of impactful pro environmental behavior (e.g., engaging in community‐based climate actions, contributing to social media climate campaigns). Finally, while we involved youth in the co‐development process, we did not involve teachers. Future research may benefit from adopting a participatory approach that includes both youth and teachers, to benefit from teachers’ professional expertise and speak to teachers’ needs.

## 5 Conclusion

Climate change presents an unprecedented threat to human development and wellbeing (IPCC 2023). Environmental education can play a critical role in preparing youth for a challenging future—and in equipping them to help create a brighter one (UNESCO 2020). However, educational and developmental science approaches to developing environmental education for youth have remained largely siloed (Sanson et al. 2018). In the present research, we found that using a developmentally‐informed approach to designing educational activities can enhance the efficacy of real‐world environmental education, illustrating the benefits of bridging this disciplinary divide. We hope that our research can promote the development of effective, engaging environmental education for youth and inspire further work to support human development in the face of climate change.

## Funding

This research has received funding from the European Research Council (ERC) under the European Union's Horizon 2020 research and innovation program (grant agreement No 864137 awarded to Sander Thomaes).

## Ethics Statement

This research received approval from a local ethics board (Utrecht University 23‐0022 and 24‐0036).

## Conflicts of Interest

We have no conflicts of interest to disclose.

## Supporting information




**Supporting File 1**: desc70197‐sup‐0001‐SupMat.docx

## Data Availability

The data and code needed to reproduce our results are available on the Open Science Framework (https://osf.io/jc8e3).
