# Supplementary material for: Using Motive‐Alignment to Enhance Environmental Education for Youth: A Participatory Field Experiment
Source: Dev Sci. 2026 Apr 23;29:e70197. doi: 10.1111/desc.70197 (PMC13106912; doi:10.1111/desc.70197)
Supplement: Supplementary file 1 — Supporting File 1: desc70197‐sup‐0001‐SupMat.docx [file DESC-29-e70197-s001.docx]

**Supplementary Materials for**

**“Using Motive-Alignment to Enhance Environmental Education for Youth:**

**A Participatory Field Experiment”**

**Overview**

- Supplement 1: Description of environmental education interventions
- Supplement 2: Exploratory measures
- Supplement 3: Associations among covariates and outcome variables
- Supplement 4: Confirmatory frequentist analyses
- Supplement 5: Sensitivity analyses
- Supplement 6: Exploratory analyses

**Supplement 1: Description of environmental education interventions**

**Table S1.**

*Activities per Condition*

| *Activity 1 (in both Control and Motive-Alignment Inspired Conditions)* | |
| --- | --- |
| Introduction and Getting to Know Each Other (10 min.)   - Guest teachers introduce themselves and explain that they are there to give a lesson about climate change on behalf of the Youth Climate Movement and to kick off the Trees for Schools program at their school - Teachers provide brief overview of the lesson and how this lesson fits into the Trees for Schools program - Warm-up activity for students: Students presented with set of statements and asked to raise their hand if the statement is true for them (e.g., “I have a pet”) | |
| *Activity 2 (in both conditions)* | |
| Causes & Consequences of Climate Change (15 min.   - Introduction to what climate change is through a 3-minute animated video - Small group activity (3-5 students): each group receives a stack of labelled photos of “causes” (e.g., increased meat consumption) and “consequences” of climate change (e.g., more extreme weather events) - Students given the challenge of creating chains linking the elements in the stack (e.g., airplane travel 🡪 more CO2 in atmosphere 🡪 heatwaves 🡪 wildfires 🡪 less CO2 absorption) - Class-wide reflection on what students thought of the activity | |
| *Activity 3 (Control Condition)* | *Activity 3 (MA-Inspired Condition)* |
| Fact & Opinion: What Do We Hear about Climate Change? (15 min.)   - Teachers show examples of headlines about climate change and young people in the media commenting on climate change, reflection on how climate change is presented in the media - Activity: Is it a fact or an opinion? - Students vote on whether statements are facts or opinions (e.g., Dutch is our national language; Windmills are the solution to using less fossil fuels) - Class-wide discussion on the difference between a fact and an opinion | Young People Taking Action on Climate Change (15 min.)   - Teachers show examples of young people trying to address climate change (same slide as in Control, different focus) - Teachers emphasize high levels of awareness among youth and that they are increasingly taking action in their own lives - Activity: students reflect in pairs for 5 minutes on the questions: Why are young people eager to address climate change? Why are young people better able to promote sustainable change than our parents’ generation? - Class-wide discussion of the questions and the answers that came up in pairs |
| *Activity 4 (in both conditions)* | |
| Collaborating Across Diverse Opinions and Values (20 min.)   - Reflection on how individuals have and form their own opinions based on their values and experiences - Example of two individuals with different perspectives on single-use plastics: an owner of a restaurant and a forest ranger. One benefits, the other is critical. - Activity: Plan a hypothetical barbeque for the class, with all students receiving a different identity card containing a set of values and opinions they need to bargain for in the planning of the barbeque (the class has a hypothetical budget of 50 euros) - Students have time to discuss and come up with a plan, followed by a reflection on how the planning went and what it was like to collaborate across different perspectives and goals. Reflection on how this activity relates to politics and the difficulties of addressing climate change | |
| *Activity 5 (Control Condition)* | *Activity 5 (MA-Inspired Condition)* |
| What You Can Do (in the Workforce) (15 min.)   - Explanation of how individuals, the government, and business influence one another - Explanation of the “snowball effect” and how young people can make a difference as consumers, citizens, and workers - Activity: Each student receives three posits and writes down their answer to three reflection questions to reflect on how they can contribute in their future work (1. What is already happening in your field around sustainability? 2. What sustainability changes can you think of for your work environment? 3. In what areas is there the most room for improvement?) | What You Can Do (Based on Personal Values) (15 min.)   - Explanation of how individuals, the government, and business influence one another - Explanation of the “snowball effect”, how young people can make a difference in diverse roles, and how they benefit from their actions (e.g., can make an impact and showcase their values) - Activity: Each student receives three posits and writes down their answer to three reflection questions (1. Quite a few young people who stand up for a sustainable world are respected by peers. Why is that so, do you think? 2. How can you care for a sustainable world in a way that suits who you are? 3. Why do you think it is important for young people to be able to contribute to a sustainable world?) |

**Supplement 2: Exploratory measures**

**Experienced motive-alignment during the lesson.** We measured whether participants felt like pro-environmental engagement aligned with youth motivations for autonomy, peer status, and contribution using 3 items developed for this study, inspired by Grapsas et al. (2023). Participants reported whether, during the lesson, they felt “that standing up for the climate fits with who you are”, “respect for young people who stand up for the climate”, “that young people can make a difference and contribute to a sustainable future” using a sliding scale from 0 (*not at all*) to 100 (*very much*). We computed the mean of all items (Cronbach’s ɑ = .85).

**Environmental efficacy.** We used 6 items from the Environmental Efficacy Scale (Ojala, 2012) to measure participants’ belief that they could, through engaging in individual and collective actions, effectively contribute to reducing and preventing the negative impacts of climate change. On a 5-point Likert scale (1 = *Strongly disagree*, 5 = *Strongly agree*), participants responded to statements such as, “I think that I myself can contribute to help reduce climate change” (individual efficacy), and “I think that we together can do something to help counter climate change” (collective efficacy). Two negatively-worded scale items (i.e., “I think that I myself can do little to help counter global warming” and “I think that we together can do little to help counter global warming”) reduced internal consistency of the scale (Cronbach’s ɑ = .70). We thus computed the scale mean based on the four positively-worded items (Cronbach’s ɑ = .85) and evaluated the 6-item measure as a sensitivity analysis.

**Supplement 3: Associations among covariates and outcome variables**

Table S2 presents correlations among the primary outcome variables and potential covariates. In the post-test survey, we also measured participants’ perceptions of the guest teachers as need-satisfying in their teaching of the lesson about climate change to include as potential covariate. For this measure, we used 3 study-developed items: *The guest teachers made me feel that I can have a positive impact on climate* *change* (supporting competence); *The guest teachers made me feel that I can form my own opinion about climate change* (supporting autonomy); *The guest teachers were nice* (supporting relatedness). Items were scored using a 5-point Likert scale (1 = totally disagree, 5 = totally agree). We computed the scale score as the average across items (*M* = 2.54, *SD* = 0.58, Cronbach’s α = .73).

**Table S2.**

*Correlations among Study Variables*

| Variable | 1 | 2 | 3 | 4 | 5 |
| --- | --- | --- | --- | --- | --- |
| 1. Pro-Env. Voting T0 | – |  |  |  |  |
| *N* |  |  |  |  |  |
| 2. Pro-Env. Voting T1 | 0.71 *** | – |  |  |  |
| *N* | 334 |  |  |  |  |
| 3. Pro-Env. Voting T2 | 0.61 *** | 0.73 *** | – |  |  |
| *N* | 337 | 307 |  |  |  |
| 4. Eco-Team Engagement | 0.21 *** | 0.24 *** | 0.24 *** | – |  |
| *N* | 416 | 381 | 395 |  |  |
| 5. Age | 0.13 ** | 0.07 | 0.11 * | 0.11 * | – |
| *N* | 410 | 334 | 335 | 410 |  |
| 6. Teacher Need-Sat. | 0.24 *** | 0.32 *** | 0.32 *** | 0.03 | 0.02 |
| *N* | 312 | 354 | 289 | 355 | 312 |

*Note*. Pro-Env. Voting = Pro-environmental voting intentions. T0, T1, and T2 refer to pre-test, post-test, and two-week follow-up, respectively. Teacher Need-Sat. = students’ perceptions of guest teachers as need-satisfying in their teaching of the lesson about climate change

Following our pre-registration, we explored the effects of gender, age, school, and perceptions of the guest teachers on participants’ environmental outcomes, to determine whether these variables should be included as covariates in sensitivity analyses (see Table S3 for results).

**Table S3.**

*Predictors of Environmental Outcomes*

| Predictor | Outcome | Estimate | SE | *t* | *p* |
| --- | --- | --- | --- | --- | --- |
| Age | Eco-team engagement | 0.09 | 0.05 | 2.05 | 0.040* |
| Age | Pre-test voting intentions | 1.34 | 0.5 | 2.68 | 0.008** |
| Age | Post-test voting intentions | 0.73 | 0.59 | 1.24 | 0.216 |
| Age | Follow-up voting intentions | 1.09 | 0.53 | 2.05 | 0.041* |
| Gender | Eco-team engagement | -0.2 | 0.28 | -0.71 | 0.480 |
| Gender | Pre-test voting intentions | -0.22 | 2.69 | -0.08 | 0.934 |
| Gender | Post-test voting intentions | -4.35 | 3.06 | -1.42 | 0.157 |
| Gender | Follow-up voting intentions | -2.41 | 2.98 | -0.81 | 0.419 |
| School (2 vs. 1) | Eco-team engagement | -0.45 | 0.26 | -1.71 | 0.088 |
| School (3 vs. 1) | Eco-team engagement | -0.45 | 0.27 | -1.68 | 0.093 |
| School (2 vs. 1) | Pre-test voting intentions | 2.37 | 2.76 | -0.86 | 0.391 |
| School (3 vs. 1) | Pre-test voting intentions | -3.83 | 2.73 | -1.4 | 0.162 |
| School (2 vs. 1) | Post-test voting intentions | -1.01 | 2.84 | -0.35 | 0.723 |
| School (3 vs. 1) | Post-test voting intentions | -3.28 | 2.9 | -1.13 | 0.260 |
| School (2 vs. 1) | Follow-up voting intentions | 1.4 | 2.8 | 0.5 | 0.617 |
| School (3 vs. 1) | Follow-up voting intentions | -8.56 | 2.76 | -3.1 | 0.002** |
| Perceptions of guest teachers | Eco-team engagement | 0.13 | 0.13 | 0.98 | 0.329 |
| Perceptions of guest teachers | Pre-test voting intentions | 6.18 | 1.4 | 4.42 | < .001*** |
| Perceptions of guest teachers | Post-test voting intentions | 7.81 | 1.25 | 6.27 | < .001*** |
| Perceptions of guest teachers | Follow-up voting intentions | 7.76 | 1.36 | 5.7 | < .001*** |

Given these results, we conducted sensitivity analyses including age as a covariate in the analyses concerning eco-team engagement and including age, school, and perceptions of the guest teachers as covariates in the analyses concerning pro-environmental voting intentions (see Supplement 4).

**Supplement 4: Confirmatory frequentist analyses**

Eco-Team Engagement: When adopting a frequentist approach to our confirmatory analyses concerning eco-team engagement, we found that the conditions did not meaningfully differ with respect to encouraging eco-team engagement (which is similar to what we concluded based on the Bayesian approach; see Table S4).

**Table S4. Frequentist Mixed-Effects Ordinal Regression: Eco-Team Engagement by Condition**

| Effect | Estimate | *SE* | *z* | *p* |
| --- | --- | --- | --- | --- |
| Fixed effects |  |  |  |  |
| Intercept [0\|1] | 1.58 | 0.22 | 7.31 |  |
| Intercept [1\|2] | 2.95 | 0.27 | 11.12 |  |
| Intercept [2\|3] | 3.37 | 0.29 | 11.54 |  |
| Condition | 0.33 | 0.28 | 1.18 | .240 |
| Random effects | Variance | Std. Dev. |  |  |
| Class ^a^ | 0.22 | 0.47 |  |  |

*Note*. The intercepts are thresholds representing the log-odds of being in a certain category of the outcome variable (e.g., Intercept 0|1 = odds of no interest (0) vs. interest (1) at post-lesson). Condition 0 = control; 1 = motive-alignment.

^a^ Number of observations at the class level = 31.

Change in Pro-Environmental Voting Intentions: When adopting a frequentist approach to our confirmatory analyses concerning change in pro-environmental voting intentions, we found that youth who received the modified lesson exhibited a greater increase in pro-environmental voting intentions over time compared to youth who received the original lesson. Both groups exhibited an increase in pro-environmental voting intentions from pre-test to post-test, but the modified lesson resulted in a greater increase both at post-test and at follow-up (see Table S5).

**Table S5. Frequentist Mixed-Effects Linear Regression: Change in Pro-Environmental Voting Intentions by Condition**

| Effect | Estimate | *Std. Error* | *t* (*df*) | *p* |
| --- | --- | --- | --- | --- |
| Fixed effects |  |  |  |  |
| Intercept | 51.27 | 1.45 | 35.46 (36.92) | < .001 |
| Time 1 | 1.14 | 1.37 | 0.83 (749.50) | .408 |
| Time 2 | -0.95 | 1.35 | -0.71 (755.80) | .480 |
| Time 1 * Condition | 4.21 | 1.83 | 2.30 (687.77) | .022 |
| Time 2 * Condition | 3.74 | 1.81 | 2.07 (683.29) | .039 |
| Random effects | Variance | Std. Dev. |  |  |
| Individual ^a^ | 28.17 | 5.31 |  |  |
| Class ^b^ | 347.60 | 18.64 |  |  |

*Note*. Time 1 = dummy coded to represent post-test, compared to pre-test. Time 2 = dummy coded to represent follow-up, compared to pre-test. Condition 0 = control; 1 = motive-alignment.

^a^ Number of observations at the individual level= 490.

^b^ Number of observations at the class level = 31.

**Supplement 5: Sensitivity analyses**

Eco-Team Engagement Including Age as Covariate: When including age as a covariate in our confirmatory analyses concerning eco-team engagement, we found that the conditions did not meaningfully differ with respect to encouraging eco-team engagement (BF = 1.49; PMP = .598; see Table S6 for Bayesian, and Table S7 for frequentist, results).

**Table S6. Bayesian Mixed-Effects Ordinal Regression: Eco-Team Engagement by Condition including Age as Covariate**

| Effect | Estimate | *Est. Error* | 95% CI | |
| --- | --- | --- | --- | --- |
|  |  |  | *LL* | *UL* |
| Fixed effects |  |  |  |  |
| Intercept [0\|1] | 2.85 | 0.93 | 1.00 | 4.67 |
| Intercept [1\|2] | 4.30 | 0.95 | 2.42 | 6.15 |
| Intercept [2\|3] | 4.71 | 0.96 | 2.82 | 6.58 |
| Age | 0.07 | 0.05 | -0.03 | 0.17 |
| Condition | 0.35 | 0.34 | -0.31 | 1.02 |
| Random effects |  |  |  |  |
| Class ^a^ | 0.56 | 0.22 | 0.13 | 1.01 |

*Note*. The intercepts are thresholds representing the log-odds of being in a certain category of the outcome variable (e.g., Intercept 0|1 = odds of no interest (0) vs. interest (1) at post-lesson). Condition 0 = control; 1 = motive-alignment.

^a^ Number of observations at the class level = 31.

**Table S7. Frequentist Mixed-Effects Ordinal Regression: Eco-Team Engagement by Condition including Age as Covariate**

| Effect | Estimate | *SE* | *z* | *p* |
| --- | --- | --- | --- | --- |
| Fixed effects |  |  |  |  |
| Intercept [0\|1] | 2.91 | 0.90 | 3.23 |  |
| Intercept [1\|2] | 4.36 | 0.92 | 4.73 |  |
| Intercept [2\|3] | 4.73 | 0.93 | 5.09 |  |
| Age | 0.08 | 0.05 | 1.60 | .111 |
| Condition | 0.35 | 0.31 | 1.15 | .252 |
| Random effects | Variance | Std. Dev. |  |  |
| Class ^a^ | 0.24 | 0.49 |  |  |

*Note*. The intercepts are thresholds representing the log-odds of being in a certain category of the outcome variable (e.g., Intercept 0|1 = odds of no interest (0) vs. interest (1) at post-lesson). Condition 0 = control; 1 = motive-alignment.

^a^ Number of observations at the class level = 31.

Change in Pro-Environmental Voting Intentions: When including age, school, and perceptions of the guest teachers as covariates in our confirmatory analyses concerning change in pro-environmental voting intentions, we found that youth who received the modified lesson exhibited a greater increase in pro-environmental voting intentions over time compared to youth who received the original lesson (BF = 14513.07, PMP = 1; see Table S8 for Bayesian, and Table S9 for frequentist, results). For one of the effects (condition differences in voting intentions at follow-up), the p-value dropped below the conventional significance threshold (i.e., p = .063).

**Table S8. Bayesian Mixed-Effects Linear Regression: Change in Pro-Environmental Voting Intentions by Condition including Age, School, and Perceptions of Teachers as Covariates**

| Effect | Estimate | *Est. Error* | 95% CI | |
| --- | --- | --- | --- | --- |
|  |  |  | *LL* | *UL* |
| Fixed effects |  |  |  |  |
| Intercept | 15.76 | 10.73 | -5.41 | 37.03 |
| Age | 0.71 | 0.53 | -0.34 | 1.75 |
| Teacher Need-Sat. | 6.65 | 1.23 | 4.24 | 9.07 |
| School 2 | 0.33 | 3.61 | -6.78 | 7.36 |
| School 3 | -3.97 | 3.70 | -11.40 | 3.18 |
| Time 1 | 0.85 | 1.44 | -1.99 | 3.68 |
| Time 2 | -1.27 | 1.57 | -4.32 | 1.85 |
| Time 1 * Condition | 4.75 | 1.90 | 1.02 | 8.48 |
| Time 2 * Condition | 3.79 | 2.05 | -0.23 | 7.83 |
| Random effects |  |  |  |  |
| Individual ^a^ | 18.23 | 0.91 | 16.53 | 20.08 |
| Class ^b^ | 5.02 | 2.01 | 0.86 | 8.98 |

*Note*. Teacher Need-Sat. = students’ perceptions of guest teachers as need-satisfying in their teaching of the lesson about climate change. School 2 = dummy coded to represent School 2, compared to School 1. School 3 = dummy coded to represent School 3, compared to School 1. Time 1 = dummy coded to represent post-test, compared to pre-test. Time 2 = dummy coded to represent follow-up, compared to pre-test. Condition 0 = control; 1 = motive-alignment.

^a^ Number of observations at the individual level= 312.

^b^ Number of observations at the class level = 31.

**Table S9. Frequentist Mixed-Effects Linear Regression: Change in Pro-Environmental Voting Intentions by Condition including Age, School, and Perceptions of Teachers as Covariates**

| Effect | Estimate | *Std. Error* | *t* (*df*) | *p* |
| --- | --- | --- | --- | --- |
| Fixed effects |  |  |  |  |
| Intercept | 15.76 | 10.73 |  | .143 |
| Age | 0.71 | 0.52 |  | .178 |
| Teacher Need-Sat. | 6.65 | 1.25 |  | < .001 |
| School 2 | 0.39 | 3.52 |  | .913 |
| School 3 | -3.97 | 3.63 |  | .285 |
| Time 1 | 0.84 | 1.44 |  | .558 |
| Time 2 | -1.27 | 1.55 |  | .415 |
| Time 1 * Condition | 4.75 | 1.90 |  | .013 |
| Time 2 * Condition | 3.80 | 2.04 |  | .063 |
| Random effects | Variance | Std. Dev. |  |  |
| Individual ^a^ | 328.36 | 18.121 |  |  |
| Class ^b^ | 26.28 | 12.39 |  |  |

*Note*. Teacher Need-Sat. = students’ perceptions of guest teachers as need-satisfying in their teaching of the lesson about climate change. School 2 = dummy coded to represent School 2, compared to School 1. School 3 = dummy coded to represent School 3, compared to School 1. Time 1 = dummy coded to represent post-test, compared to pre-test. Time 2 = dummy coded to represent follow-up, compared to pre-test. Condition 0 = control; 1 = motive-alignment.

^a^ Number of observations at the individual level = 312.

^b^ Number of observations at the class level = 31.

**Supplement 6: Exploratory analyses**

**Statistical Approach**

We used Bayesian informative hypothesis testing to evaluate evidence for (or against) our expectation that the motive-alignment inspired lesson, compared to the original lesson, would increase participants’ perception of pro-environmental engagement as a way to contribute meaningfully to society, gain peer status, and express autonomy.

**Results and Discussion**

Table S10 presents descriptive statistics for the full sample and by condition. Directly following the lesson, youth in the MA-inspired condition did not meaningfully differ from youth in the control condition in feeling more motive-alignment during the lesson (BF = 1.434, PMP = .589). However, they did report a heightened sense of environmental efficacy compared to youth in the control condition (BF = 5.931, PMP = .856). These results were consistent also when analyzing the 6-item measure of environmental efficacy (BF = 6.551, PMP = .868). The MA-inspired lesson thus presented pro-environmental engagement as a more effective means for youth to make an impact, aligning pro-environmental engagement with the targeted motivation of societal contribution.

**Table S10. Descriptives Statistics for Full Sample and by Condition**

| Variable | Total Sample  *M* (*SD*) | Control  *M* (*SD*) | MA-Inspired  *M* (*SD*) |
| --- | --- | --- | --- |
| Environmental Efficacy | 3.43 (0.62) | 3.38 (0.62) | 3.48 (0.63) |
| *N* | 369 | 163 | 197 |
| Experienced Motive-Alignment | 58.04 (22.16) | 57.70 (21.60) | 58.32 (22.67) |
| *N* | 377 | 171 | 206 |

*Note*. Control = Control condition; MA-Inspired = Motive-alignment inspired condition. *N*s = 369, 163, and 197 for the total sample, Control condition, and Motive-Alignment inspired condition, respectively.
